# Studying the Impact of Online Mental Health Therapy: Platform Usage Patterns and Effectiveness of Chat-Based Therapy for Anxiety

**DOI:** 10.1192/j.eurpsy.2025.1443

**Published:** 2025-08-26

**Authors:** C. Gil Lopez, A. Rez, G. Hoter Ishay

**Affiliations:** 1 Research, Ifeelonline, Madrid, Spain; 2 Research, Ono Academic College, Kiryat Ono, Israel

## Abstract

**Introduction:**

The treatment landscape for mental health has transformed significantly with the emergence of digital platforms, which offer a promising public health solution by expanding access to mental health services. Despite their potential, further research is needed to evaluate both the usability and clinical effectiveness of these online interventions.

**Objectives:**

To investigate the usage patterns of an online mental health app and the effectiveness of chat-based emotional support (ES) in reducing psychological distress and achieving therapeutic goals.

**Methods:**

The analysis included data from 3,751 users of *ifeel* app onboarded in 2023, 58% of whom were female and 42% male. Two main modalities were studied: chat-based therapy,( N= 3,170) and video therapy,( N=1,942), with some overlap as users could engage in both modalities. Key metrics for analysis included session frequency, session timing, session duration, and user demographics. The study also explored chat-based ES therapy by evaluating 113 individuals at two points in time: after one week of therapy, and after three weeks. Psychological distress levels were measured using the Kessler Psychological Distress Scale (K6), and goal attainment was measured through self-reports over the two time points.

**Results:**

Usage data showed a strong preference for chat-based therapy, with 85% of users engaging in chat therapy compared to 52% who used video therapy. Younger users, particularly those aged 25-34, were the most active in engaging with chat-based therapy. On average, chat therapy users send between 14 to 17 messages per month, whereas video therapy users take 2 sessions per month. These results underscore the higher frequency of engagement in chat therapy, particularly among younger demographics. In terms of clinical effectiveness, psychological distress was reduced significantly at both one week and three weeks of therapy. After one week of treatment, participants showed a significant reduction in distress symptoms (F(1) = 5.10, p = .03*) this was evident also after three weeks (F(1) = 24.25, p = .000***). Goal attainment showed substantial progress over time, with significant improvements in both groups (F(1) = 5.03, p = .03*; F(1) = 31.61, p = .000*), indicating that users were making meaningful strides toward their therapeutic goals.

**Conclusions:**

This study underscores the usage pattern and clear preference for chat-based therapy, particularly among younger users. Chat-based emotional support therapy was found effective in reducing psychological distress, suggesting that online platforms, especially those offering chat-based therapy, are both accessible and clinically effective. Further research should focus on refining these services to maximize clinical outcomes, adapt to diverse user demographics, and ensure long-term engagement.

**Disclosure of Interest:**

None Declared

